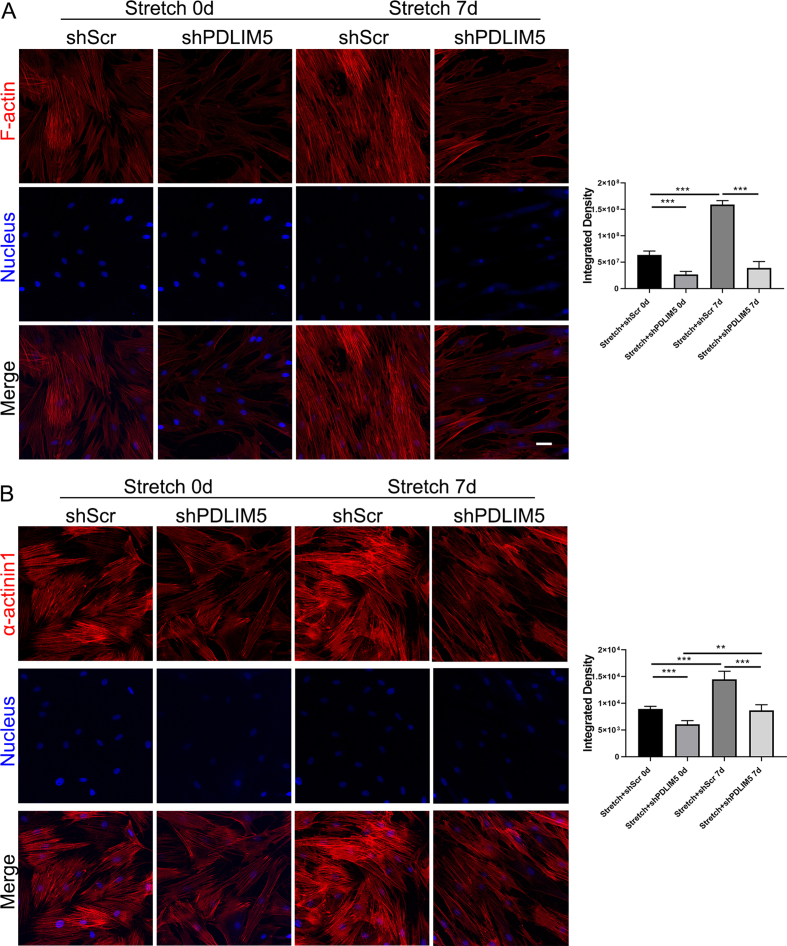# Mechanical sensor PDLIM5 promotes the osteogenesis of human adipose-derived stem cells through microfilament alterations

**DOI:** 10.1016/j.gendis.2023.06.001

**Published:** 2023-07-11

**Authors:** Yuchao Yang, Shutong Wu, Rongmei Qu, Congrong Wang, Jinyang Wang, Asmat Ullah Khan, You Pan, Wenqing Liu, Jinhui Zhu, Muhammad Akram Khan, Chujiang Xu, Jingxing Dai, Jun Ouyang

**Affiliations:** a Guangdong Provincial Key Laboratory of Medical Biomechanics & National Key Discipline of Human Anatomy, School of Basic Medical Sciences, Southern Medical University, Guangzhou, Guangdong 510515, China; b Department of Laboratory Medicine, Nanfang Hospital of Southern Medical University, Guangzhou, Guangdong 510515, China; c Department of Pathology, Guangdong Provincial People's Hospital, Guangdong Academy of Medical Sciences, Guangzhou, Guangdong 510080, China; d Department of Breast Surgery, Guangxi Medical University Cancer Hospital, Nanning, Guangxi 530000, China; e Department of Veterinary Pathology, Faculty of Veterinary and Animal Sciences, PMAS Arid Agriculture University, Rawalpindi 46300, Pakistan; f Department of Orthopedics, TCM-Integrated Hospital, Southern Medical University, Guangzhou, Guangdong 510000, China

Bone regeneration is a multifaceted, abstract, and well-coordinated physiological progression of bone formation that participates in continuous regeneration and remodeling throughout life. However, when it comes to complex clinical situations requiring extensive bone regeneration, such as massive bone defects caused by injuries, infection, or tumor removal, traditional methods do not often yield good treatment strategies or protocols due to their limitations.^1^ Stem cells, which have been shown to differentiate into diverse lines, have great potential to treat many diseases.^2^ The stem cells provide an excellent opportunity to study local strategies for bone healing and even systematic enhancement of bone repair, thereby overcoming the limitations of current methods. PDZ and LIM domain 5 (PDLIM5) is regarded as a cytoskeleton-related protein that is intimately connected to the dynamic alterations of microfilaments and can mediate signal transmissions between the cell nucleus and the cytoskeleton. PDLIM5 is also defined as a mechanosensitive protein that exhibits tension-dependent nuclear translocation in cells, displaying a mechanoconductive relationship with Yes-associated protein (YAP).^3^ Therefore, it is useful to further understand PDLIM5's molecular mechanism in osteogenic differentiation to study how it transfers external mechanical stimulation to the nuclear skeleton through the cytoskeleton and controls osteo-genes.

In this study, bioinformatics analysis of proteomic results revealed a total of 998 differentially expressed proteins, with 737 of these proteins being up-regulated and 261 being down-regulated, respectively. After two weeks of osteogenic differentiation, the expression of PDLIM5 in the osteogenic medium (OS) group was 1.523 times that in the growth medium (GM) group (Fig. 1A; Supplemental File 1). According to Gene Ontology analysis, PDLIM5 protein is substantially enriched in the binding, adhesion, and protein components of the cell matrix and plays an important role in osteogenic differentiation (Fig. 1B, C). Even though boxplot and principal component analysis plots did not reveal a statistically significant difference between the OS and GM groups, the changes in osteo-related protein levels in the OS group were more pronounced (Fig. S1A, B). Furthermore, the relationship between positive and negative genes related to osteogenic differentiation was indicated by the volcano map of the univariate Cox proportional risk regression analysis, and the PDLIM5 protein level was in the up-regulated range for promoting osteogenic differentiation (Fig. 1D). We learned from the STRING (protein–protein association networks and functional enrichment analyses) database^4^ that PDLIM5 is closely related to cytoskeletal actin and cell motor behavior (Fig. S1C), which provides evidence to support the notion that PDLIM5 is a mechanically sensitive element in terms of osteogenic differentiation.

**Figure 1:**
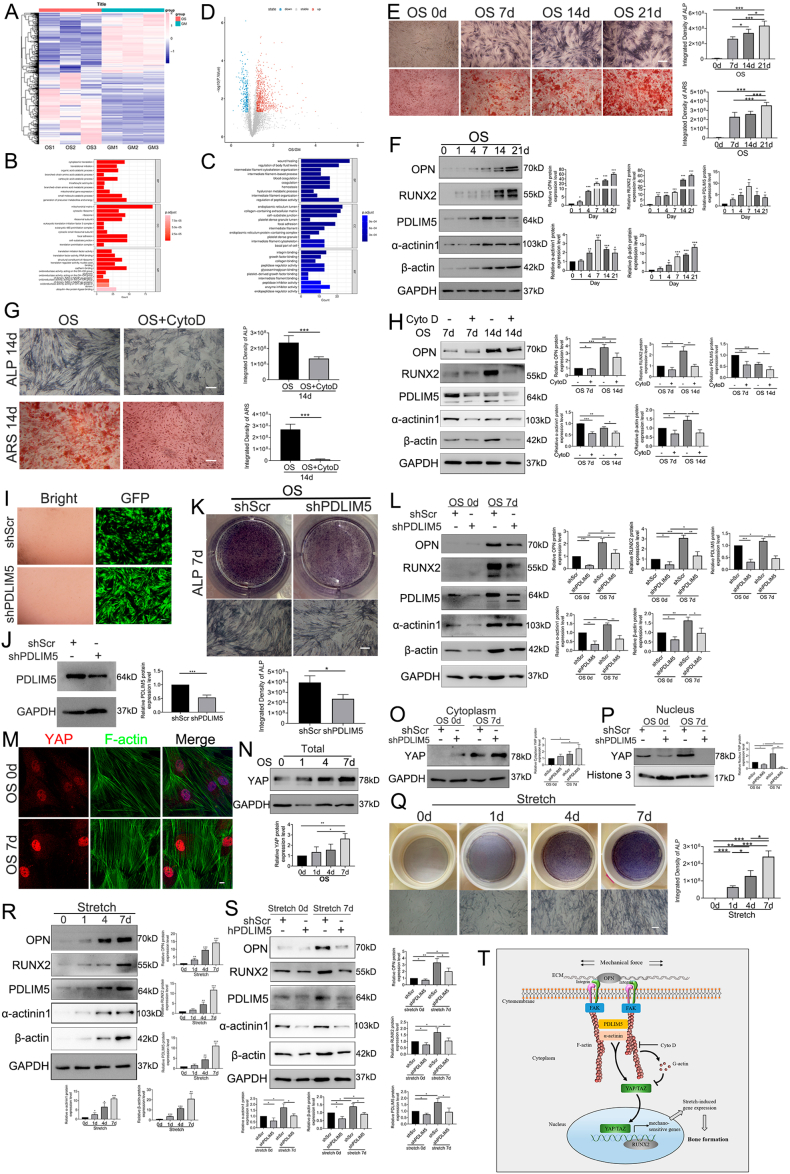
Mechanotransduction of PDLIM5 in osteogenic differentiation of hASCs by microfilament alteration. **(A)** The heatmap showing the 998 differentially expressed genes. GM: cells treated with growth medium; OS: cells treated with osteogenic differentiation medium. *P* < 0.05, log (fold change) > 1.2. **(B, C)** GO analyses showed that information of positive (A) and negative (B) osteogenic-related genes had high enrichment in biological processes (BP), cellular components (CC), and molecular functions (MF). **(D)** The volcano plot showing the gene information between the positive and negative osteogenic-related genes. **(E)** ALP staining and Alizarin Red S staining analysis with quantification of hASCs differentiation into osteogenesis. Scale bar, 200 μm. **(F)** Western blotting detection of OPN, RUNX2, PDLIM5, α-actinin 1, β-actin, and GAPDH. Densitometric quantification of the Western blotting bands normalized to GAPDH. **(G)** Osteogenic differentiation of hASCs treated with CytoD was determined by ALP staining and Alizarin Red S staining at 14 days. Scale bar, 200 μm. **(H)** Effects of CytoD on the expression of hASCs osteogenic-related protein and PDLIM5 by Western blotting analysis. **(I, J)** hASCs fluorescence intensity and protein expression of PDLIM5 by Western blotting after lentivirus infection. **(K)** ALP staining showed that PDLIM5 knockdown affected early osteogenic differentiation in hASCs. Scale bar, 200 μm. **(L)** Osteogenic protein markers (OPN, RUNX2) and expression levels of microfilament-associated proteins (α-actinin 1, PDLIM5, and β-actin) were detected by Western blotting. **(M)** The expression of nuclear YAP in hASCs osteogenic differentiation was determined by immunofluorescence at 7 days. Scale bar, 10 μm. **(N)** Total YAP protein expression in the osteogenic medium of hASCs at 0, 1, 4, and 7 days was detected by Western blotting. **(O, P)** The expression of YAP in the cytoplasm and cell nucleus with or without PDLIM5 knockdown in hASCs at 7 days was detected by Western blotting. Densitometric quantification of the Western blotting bands normalized to histone 3. **(Q)** ALP staining after 0, 1, 4, and 7 days of cyclic stretching stimulation (on a Flexcell special 6-well plate). Scale bar, 200 μm. **(R)** The expression levels of OPN, RUNX2, PDLIM5, α-actinin 1, β-actin, and GAPDH under cyclic stretching stimulation. **(S)** PDLIM5 knockdown attenuated mechanical strain-induced osteogenesis by inhibiting both osteogenic protein markers (OPN, RUNX2) and microfilament-associated proteins (α-actinin 1, PDLIM5, and β-actin). **(T)** Schematic diagram of PDLIM5 as a mechanosensitive protein that conveys mechanical signals in promoting hASCs osteogenic differentiation.

Flow cytometry was conducted to identify human adipose-derived stem cells (hASCs) typical surface markers, such as CD45, CD24, CD29, CD90, and CD105 (Fig. S2A). We used alkaline phosphatase (ALP) staining, Alizarin Red S (ARS) staining (Fig. 1E), and Western blotting (Fig. 1F) to detect the osteogenic-differentiation ability of hASCs and the expression of osteogenic markers (*e.g.*, osteopontin, OPN; RUNT-related transcription factor 2, RUNX2). Meanwhile, the expression of cytoskeletal protein microfilaments (β-actin and α-actinin 1) (Fig. 1F) also improved with the prolongation of the osteogenic differentiation time, consistent with findings from our previous work.^5^ Immunofluorescence additionally demonstrated that PDLIM5 co-localized with microfilaments (Fig. S2B, C).

When hASCs were treated with microfilament-polymerization inhibitor, cytochalasin D (CytoD) alterations in osteogenic-related markers and cytoskeletal proteins were observed. The intensities of both ALP staining and ARS staining were decreased by CytoD treatment (Fig. 1G). Western blotting results showed that the expression of the osteogenic markers OPN and RUNX2 and cytoskeletal protein microfilaments (β-actin) decreased on days 7 and 14 after CytoD treatment in comparison to the normal induced medium effects. Additionally, there was a reduction in the expression of the proteins that bind to PDLIM5 and α-actinin 1 (Fig. 1H), and there was also a marked reduction in the amount of co-localization with α-actinin 1 (Fig. S3A). PDLIM5 expression floats around in the cytoplasm as a cloud rather than being firmly bound to cytoskeletal proteins (Fig. S3B). This suggests that the cytoskeleton functions as a mechanical signaling stress fiber network that transmits signals. When polymerization is blocked, the related mechanical signal-sensitive proteins, such as PDLIM5, lose their physiological abilities to detect mechanical information and deliver mechanical signals.

In addition, we determined that PDLIM5 was down-regulated by lentivirus transfection (Fig. 1I, J; Fig. S4A, B), and its effect on hASCs behavior was verified. PDLIM5 knockdown significantly reduced the proliferation rate of hASCs, as demonstrated by cell counting kit-8 assay (Fig. S4C). Meanwhile, migration and wound-healing experiments revealed a significant downward trend after PDLIM5 downregulation (Fig. S4D, E), indicating that PDLIM5 is also involved in the hASCs growth cycle and daily activities and plays an important role in regulating hASCs cellular behaviors.

The intensity of ALP staining and the expression of the osteogenic markers OPN and RUNX2 were also significantly decreased compared with those in the non-knockdown group (Fig. 1K, L). Simultaneously, similar to what was observed among hASCs treated with CytoD, the expression of associated cytoskeletal proteins β-actin and α-actinin 1 was also significantly diminished after PDLIM5 knockdown (Fig. 1L). As demonstrated by immunofluorescence results (Fig. S5A), PDLIM5 knockdown in hASCs resulted in short rods and discontinuous microfilaments after seven days of osteogenic differentiation compared with the control cells. These results indicate that PDLIM5 plays a crucial role in regulating the expression and stability of microfilaments during osteogenic differentiation.

When cultured in the osteogenic medium environment for seven days, the distribution of YAP in the nucleus gradually increased (Fig. 1M, N). PDLIM5 knockdown triggered an increase in YAP expression in the cytoplasm, while nuclear YAP protein expression was significantly diminished on the seventh day of osteogenic differentiation (Fig. 1O, P). Immunofluorescence staining showed that nuclear translocation of YAP in the negative control group was evident and obvious, while YAP expression in the cell nucleus was drastically decreased after PDLIM5 knockdown (Fig. S5B).

Under normal medium conditions, ALP expression was induced by continuous cyclic stretching stimulation, and the amount of ALP staining gradually increased (Fig. 1Q). The expression levels of osteogenic markers OPN and RUNX2 as well as those of the cytoskeletal-related proteins β-actin, α-actinin 1, and PDLIM5 increased gradually following seven days of constant stretching stimulation (2 h/day) (Fig. 1R). Following PDLIM5 knockdown, the effect of cyclic stretching stimulation on the osteogenesis effect of hASCs was reduced (Fig. 1S). Immunofluorescence supported a similar pattern, showing that, following PDLIM5 down-regulation, the fluorescence intensities of PDLIM5, microfilaments, and α-actinin 1 were all drastically decreased (Fig. S6A, 7A, 7B). In the PDLIM5-knockdown group, nuclear YAP expression was reduced, and immunofluorescence results demonstrated that nuclear YAP expression was considerably altered in response to cyclic stress stretching (Fig. S6B).

In conclusion, mechanical stress can regulate actin stress fibers by enhancing the binding and co-localization of the PDLIM5 and α-actinin 1 proteins. The status of YAP in the cell nucleus can activate the nuclear transcription factor RUNX2 and regulate hASCs osteoblast development (Fig. 1T). It provides theoretical support for promoting fracture healing or bone defect repair with cyclic stretching stimulation in the clinic.

## Author contributions

J.O., J.D., and C.X. conceived and designed the experiments. Y.Y., S.W., C.W., R.Q., J.T., J.W., W.L., and J.Z. performed the experiments. Y.Y., Y.P, C.X., J.D., and J.O. analyzed the data and wrote the paper. Y.Y., A.U.K., Y.P., M.A.K., C.X., J.D., and J.O. revised the manuscript. The authors read and approved the final manuscript. All data were generated in-house, and no paper mill was used. All authors agree to be accountable for all aspects of this work, including ensuring its integrity and accuracy.

## Conflict of interests

The authors declare that no conflict of interests exists.

## Funding

This work was supported by the National Key R&D Program of China (No. 2022YFF1202603), the Guangdong Basic and Applied Basic Research Foundation (No. 2021A1515110440), the National Natural Science Foundation of China (No. 82002779), the Guangxi Provincial Natural Science Foundation of China (No. 2019GXNSFAA245083), the China Postdoctoral Science Foundation (No. 2022M710853), and the President's Foundation of the TCM-integrated Hospital of Southern Medical University (No. 1202103001).

## Figures and Tables

**Fig. S1:**
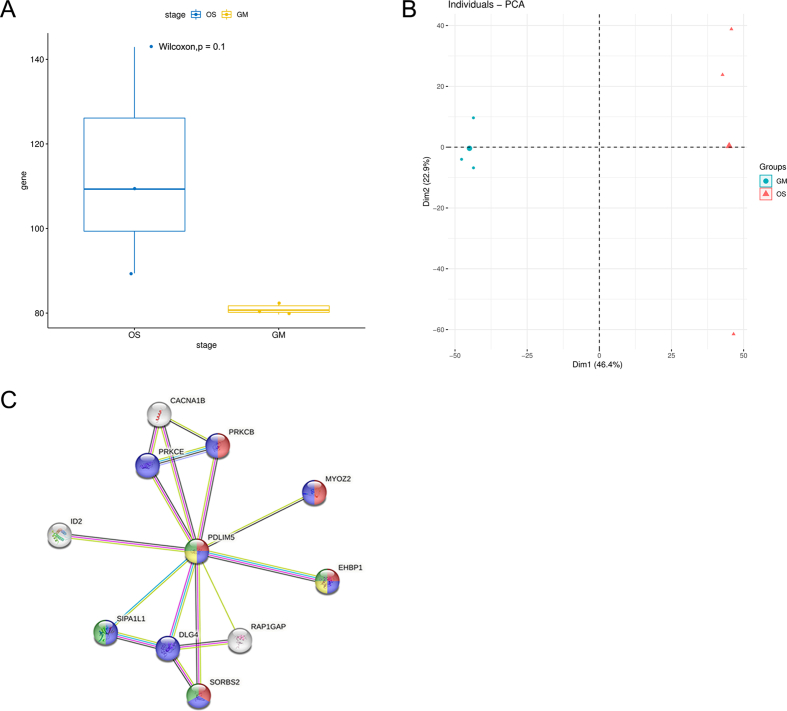


**Fig. S2:**
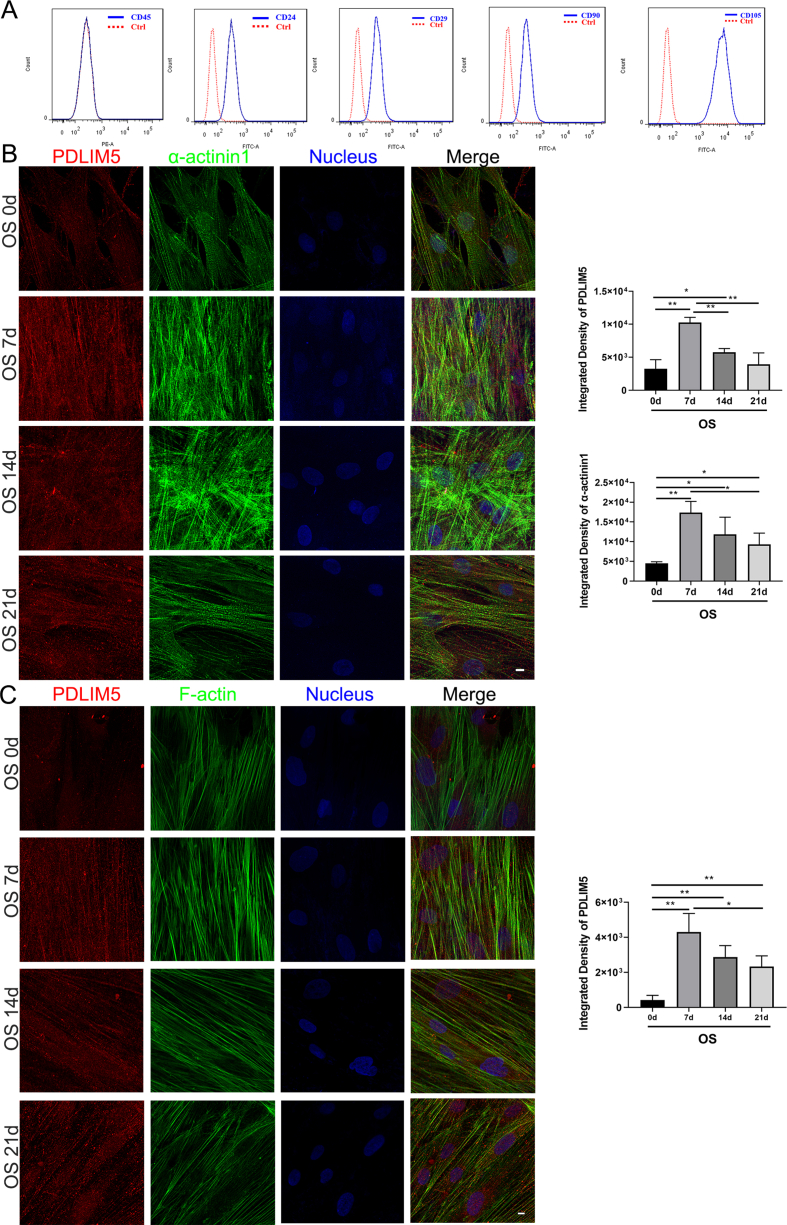


**Fig. S3:**
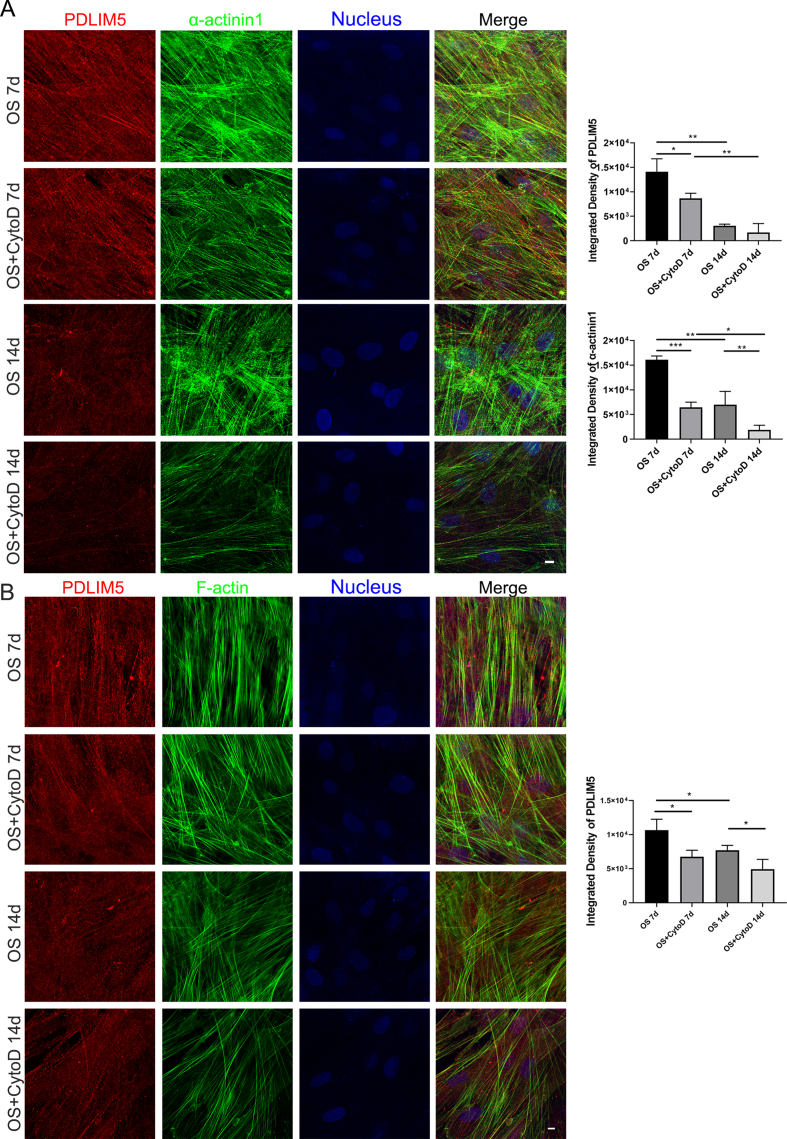


**Fig. S4:**
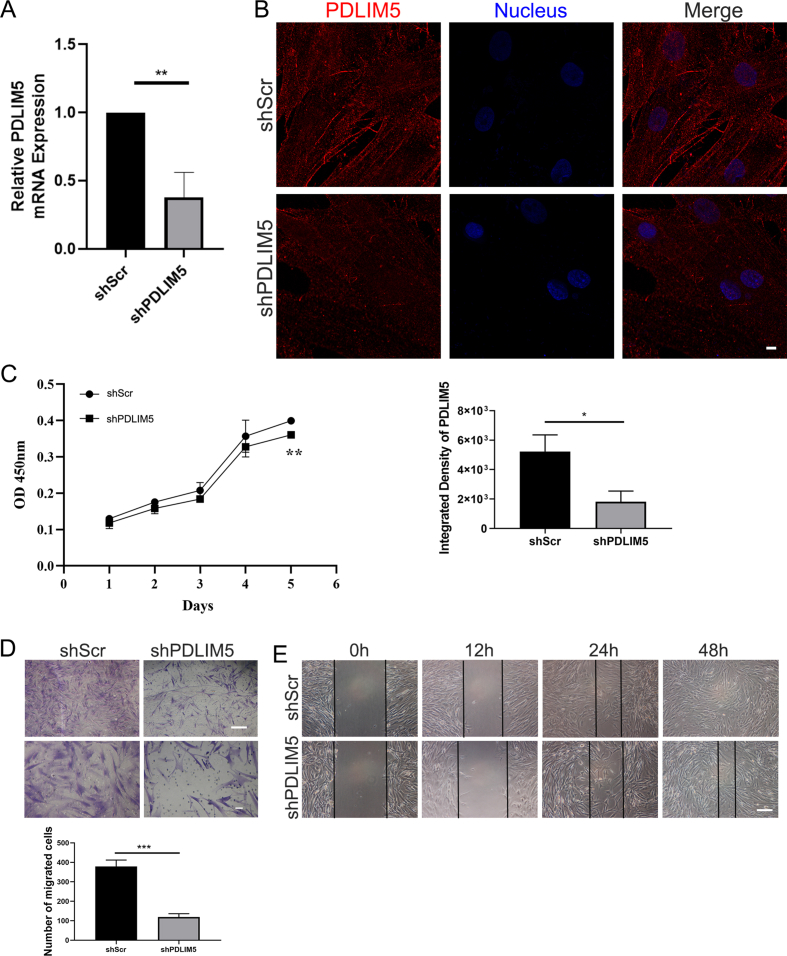


**Fig. S5:**
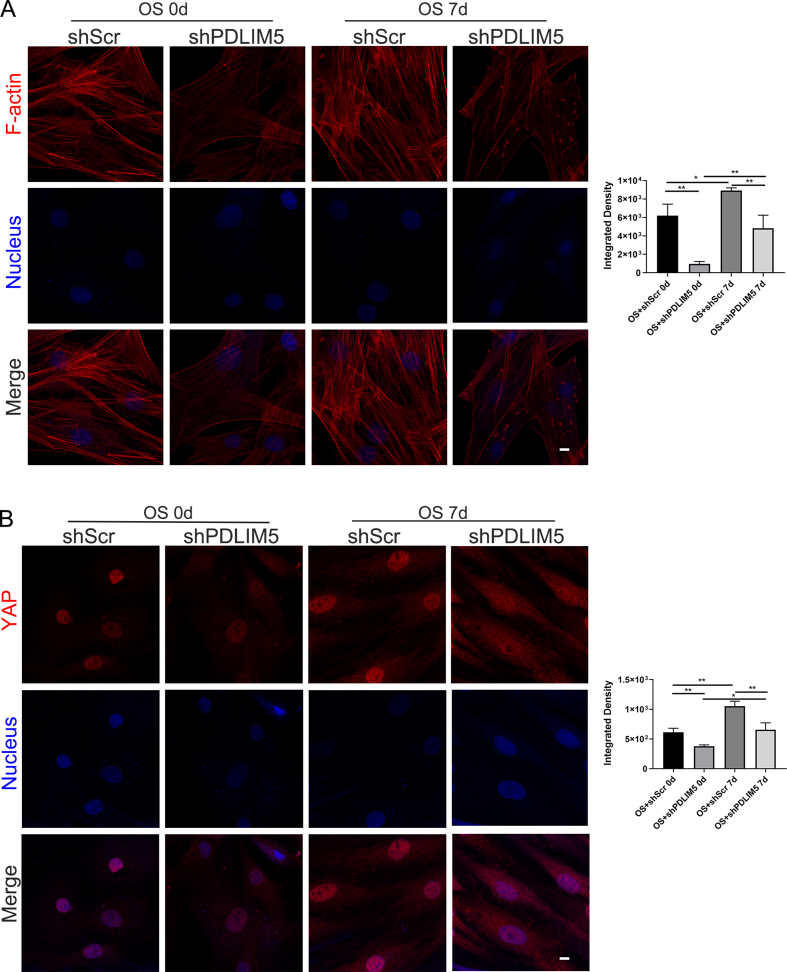


**Fig. S6:**
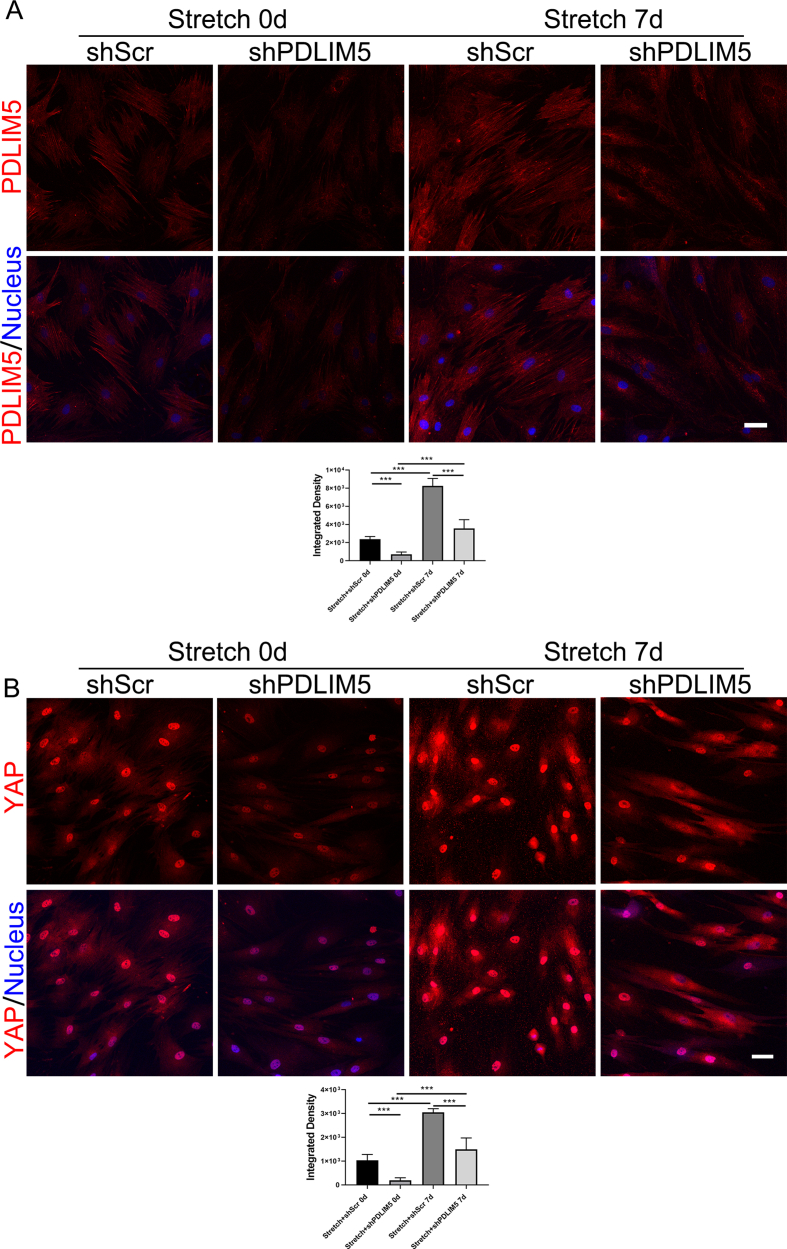


**Fig. S7:**