# Supplementary material for: Mechanical sensor PDLIM5 promotes the osteogenesis of human adipose-derived stem cells through microfilament alterations
Source: Genes Dis. 2023 Jul 11;11(3):101023. doi: 10.1016/j.gendis.2023.06.001 (PMC10828594; doi:10.1016/j.gendis.2023.06.001)
Supplement: Multimedia component 1 [file mmc1.docx]

**Materials and Methods**

**Reagents**

Antibodies (Abs) used in this study include osteopontin (OPN, Abcam, Cambridge, UK), Runt-Related Transcription Factor 2 (RUNX2, CST#12556S, Danvers, MA, USA), PDLIM5 (ab85967, for western blot) & (H00010611-M01, Abnova Company, Taipei, Taiwan, for immunofluorescence), α-actinin 1 (ab68194), β-actin (CST#4970S), glyceraldehyde-3-phosphate dehydrogenase (GAPDH, AP0063, Bioworld, Bloomington, MN, USA), YAP (CST#14074S), Histone H3 (ab5103), CD24 (555428, BD Bioscience, San Jose, CA, USA), CD29 (552828, BD Bioscience), CD90 (555593, BD Bioscience), CD45 (bs-10602R, Bioss Antibodies, Woburn, MA, USA), and CD105 (bs-4609R, Bioss Antibodies). Fluorescent-conjugated phalloidin, secondary antibody and DAPI were from Thermo Fisher Scientific. Cytochalasin D (CytoD, 0.1 μg/mL, ab143484, abcam) was from Abcam. Osteogenic differentiation medium (OS) containing 10% fetal bovine serum (FBS, Gibco, Waltham, MA, USA), 1% penicillin/streptomycin (Gibco), dexamethasone (100 nM, D4902, Sigma-Aldrich, St. Louis, MO, USA), L-Ascorbic acid (37.5 mg/L, V900134, Sigma-Aldrich), β-glycerophosphate sodium (10 mM, G9422, Sigma-Aldrich) and Vitamin D3 (10 nM, CAS:67-97-0, Solarbio, Beijing, China).

**Isotopic multi-tag relative quantitative proteomics (iTRAQ) experiments and bioinformatics analysis**

iTRAQ quantitative proteomic analysis was performed using high-resolution mass spectrometer Q Exactive Plus (Thermo Scientific), and iTRAQ peptide labeling, classification, mass spectrometry analysis, database comparison and bioinformatics analysis were performed on the qualified samples. Proteome Discoverer 2.1 software was used to convert the original atlas file (.raw format) produced by Q Exactive Plus into .mgf format and submit it to MASCOT 2.5 server for database retrieval. Then, the file retrieved on MASCOT server (.dat format) is passed back through Proteome Discoverer 2.1, and the result with high confidence is obtained according to the screening criterion of FDR < 0.01, and bioinformatics analysis was performed.

**Cell culture, identification and transfection**

Human ASCs were purchased from Cyagen Biosciences (HUXMD-90011, Suzhou, China). Adherent hASCs were then cultured in culture dishes with high-sugar Dulbecco’s modified Eagle’s medium (H-DMEM; Gibco, Waltham, MA, USA) consisting of 10% FBS, 1% penicillin-streptomycin (Gibco), and 0.2% plasmocin prophylactic (Invivo Gen, San Diego, CA, USA) in an incubator at 37 ℃ and 5% CO_2_. The human ASCs at passages 3–7 were used for subsequent experiments.

For flow cytometry, hASCs were digested, centrifuged and suspended, then they were incubated with the following primary antibodies (CD45, CD24, CD29, CD90, and CD105) on ice for 2 hours. After washing three times with PBS for 5 minutes each, and then incubated with the corresponding secondary antibody in the dark on ice for 1 hour. After washing with PBS for three times, BD LSR II flow cytometry was used for subsequently analysis.

To inhibit the expression of human PDLIM5, lentivirus for negative control (LVCON313) and LV-PDLIM5 (GIEL0259935) were assembled and produced by GeneChem (GeneChem Co.,Ltd, Shanghai, China) to knock down PDLIM5 expression. A pre-experiment lentiviral infection was first performed to optimize the best conditions for infection including MOI and infection time. Before transfection, the virus was diluted in fresh medium according to the transfected cell density ratio and enhancer Histrans P was mixed in solution. Subsequently, passages 3 hASCs of 7000/cm^2^ were seeded and cultured on the 6-well plate. The hASCs were transfected with the corresponding lentiviruses when the cell confluence upto 30% to 40% on the second day. Then, medium containing the virus was replaced with GM and the cells were further cultured. Subsequent changes in the medium was in accordance with the cell growth status. After 72–96 hours of culturing, the cell transfection efficiency was observed under a fluorescence microscope (Olympus, Tokyo, Japan).

**Western Blotting**

Protein extracts were prepared from cells stimulated with the osteogenic induction medium and from cells induced by mechanical strain. Briefly, hASCs with different treatment were washed three times with pre-cooled PBS. Then, the cells were incubated with the cell lysis buffer from the whole protein extraction kit (Whole Cell Lysis Assay, KeyGEN Bio TECH, Nanjing, China). And, nuclear protein was extracted using the nuclear and cytoplasmic protein extraction kit (KeyGEN Bio TECH) to prepare cytoplasmic and nuclear lysate according to the manufacturer’s instructions. The adherent cells were scraped with a cell scrape. The cells were then gently agitated at 4°C on a refrigerated shaker for 30 minutes. Then, the cell lysate was centrifuged at 12,000 × *g* at 4 °C for 10 minutes. The supernatant was harvested, mixed with 5X sample loading buffer, and boiled in a 100 °C water bath for 5–10 minutes.

Equal amounts of protein samples were separated on a 10% SDS-PAGE gel, then transferred onto polyvinylidene fluoride (PVDF) membranes (Millipore, Burlington, MA). The PVDF membrane was blocked with 5% skim milk for 1 hour at room temperature, and then GAPDH (dilution 1:10000), OPN (dilution 1:1000, Rabbit IgG), RUNX2 (dilution 1:1000, Rabbit IgG), PDLIM5 (dilution 1:1000, ab85967, Rabbit IgG), α-actinin 1 (dilution 1:1000, Rabbit IgG), β-actin (dilution 1:1000, Rabbit IgG), YAP (dilution 1:1000, Rabbit IgG) and Histone H3 (dilution 1:1000, Rabbit IgG) specific antibodies were added respectively and incubated overnight at 4 °C. After that, the PVDF membranes were washed with TBST three times for 15 minutes each and incubated with horseradish peroxidase-conjugated secondary antibodies for one hour at room temperature. Subsequently, after washing the blots three times with TBST for 15 minutes each, protein bands were detected by using enhanced chemiluminescence (ECL) chromogenic substrate (FUDE Biological, Hangzhou, China). Signal intensity was assessed by using a Tanon-5500 chemiluminescence detection system (Tanon Science & Technology Ltd, Shanghai, China). The immunoreactive bands were quantitatively analyzed through Image J software (National Institutes of Health, Bethesda, MD, USA).

**RNA Isolation and** **Quantitative Real-Time PCR (qRT-PCR)**

Total RNA from the cultured hASCs was isolated and purified using Trizol (Invitrogen, Carlsbad, CA, USA) reagent according to the instructions from manufacturer. First-strand cDNAs were produced by reverse transcription RNA using a RevertAid First Strand cDNA Synthesis Kit (Thermo Scientific). qRT-PCR was executed using ABI Step One Plus System (Applied Biosystems, USA) and a fluorescence-labeled SYBR Green/ROX qPCR Master Mix Kit (Thermo Scientific). The sequences for qRT-PCR of each gene were listed in Table 1.

Table 1. Primers used in the qRT-PCR

| Gene | Forward primer (5’-3’) | Reverse primer (3’-5’) |
| --- | --- | --- |
| GAPDH  PDLIM5 | TCGGAGTCAACGGATTTGGT  TTAGTGGCACTGGGGAAATC | TTCCCGTTCTCAGCCTTGAC  GATCTTCCTTTGGCATCGAC |

**Immunofluorescence Assay**

hASCs were seeded on microscope cover glass in 24-well plates with different treatment. hASCs were washed three times in DMEM for 2 minutes each, and fixed with 3.7% paraformaldehyde for 10 minutes at room temperature. Then treated with 0.1% Triton X-100 for 10 minutes to increase membrane permeability, followed by 1 hour incubation with 2 % BSA to block non-specific effects. The cells were washed and incubated overnight at 4 °C with anti-PDLIM5 (dilution 1:500, H00010611-M01), anti-α-actinin1 (dilution 1:500, ab68194) antibodies, β-actin (dilution 1:500, CST#4970S), YAP (dilution 1:500, CST#14074S) and phalloidin (1:500, excitation/emission: 495/518 nm). After washing twice in PBS, they were further stained with a fluorescently-labeled secondary antibody, AlexFluor 488 goat anti-rabbit and AlexFluor 568 goat anti-mouse (1:500, Thermo Fisher Scientific, Waltham, MA, USA) for 1 h, and 4′,6-diamidino-2-phenylindole (DAPI, 1:500) at room temperature for 0.5 h. The stained cells were observed and photographed using a confocal laser scanning microscope (LSM 880, Carl Zeiss, Jena, Germany).

**ALP Staining and** **Alizarin Red S Staining**

In order to examine early mineralization, hASCs were stimulated in osteogenic differentiation medium (OS) in 12-well plates. Specifically, the alkaline phosphatase color development kit included 3-bromo-4-chloro-3-indolyl phosphate (BCIP)/nitro blue tetrazolium (NBT) was used for the ALP staining (Beyotime). Under an optical microscope (Olympus BX51, Tokyo, Japan), the positive area of ALP was evaluated in three randomly selected fields using the Image J software.

Alizarin red S staining (Cyagen Biosciences, Suzhou, China) was executed to assess late mineralization. hASCs were plated in 12-well plates and cultured in OS medium. After two weeks of culture, the hASCs were fixed in 3.7% paraformaldehyde at room temperature for 20 minutes and washed three times with double distilled water (ddH_2_O). Lastly, the cells were stained with ARS solution for 30 minutes and washed with ddH_2_O. An optical microscopewas used to capture the staining on three randomly selected fields of the ARS-positive area and staining area was evaluated using the Image J software.

**Cell Proliferation Assay (CCK8 Assay)**

The cells were seeded in a 96-well plate at a density of 5 × 10^3^ cells per well to assess lentivirus transduction influence on cell proliferation. To measure the proliferation of knockdown hASCs, the cells were then cultured in growth medium (GM) for 5 days. The medium was changed to 100 μL DMEM without FBS (Gibco) containing 10% CCK-8 (Dojindo, Kumamoto, Japan) for 1 h at 37°C. The absorbance of 450 nm (A_450_) was assessed using a microplate reader (Thermo Scientific, Multiskan Go, Danvers, MA, USA).

**Cell migration and wound healing**

hASCs were cultured in serum-free medium in top chambers of transwell plates (1×10^5^ cells per chamber, Corning, NY, USA). DMEM supplemented with 10% FBS served as an attractant in lower chambers. Cells were fixed with 3.7% PFA and stained with 1% crystal violet after 12–16 h. Non-migrating cells on the upper side of the membrane were gently wiped off, and the stained cells on the lower side were observed under a microscope. The number of migrating cells in five fields per chamber was counted and average values were calculated.

The cells were seeded into a 6-well plate at a density of 1 × 10^5^ cells per well and divided into two groups: shScr, sh-PDLIM5. After the cells were adherent, the monolayer of cells was scratched through each hole with the tip of a sterile 1-mL pipette gently, slowly, and gently washed with PBS to remove any detached or dead cells. Replenish the wells with fresh medium. After 12 h, 24 h and 48 h, the cells that migrated to the empty space to evaluate their motor ability by a microscope.

**Cyclic strain loading**

hASCs were cultured (1 ×10^5^ cells per well) on collagen I-coated silicone membrane plates (Bioflex, Flexcell International, NC, USA) for 2 days. The hASCs on the coated plate are applied cyclic tensile stress through the Flexcell FX-5000 system (Flexcell International). The application scheme was 10% tensile strain, 0.5 Hz, 2 h every day. CCK8 and ALP staining, western blotting, qRT-PCR, and immunofluorescence were performed immediately after the end of cyclic stretching at each time point. Cells cultured under the same conditions but not subjected to mechanical loading were used as controls.

**Statistical Analysis**

The statistical data were expressed as mean ± standard deviation of at least three independent experiments. Statistical significance was estimated using the Student’s t-test. Values of *P*＜0.05 were considered significant.

**Abbreviations**

PDLIM5: PDZ and LIM domain 5; CytoD: Cytochalasin D; ALP: alkaline phosphatase; ARS: alizarin red S; OPN: osteopontin; RUNX2: Runt-related transcription factor 2; YAP: Yes-associated protein; AMPK: AMP-activated protein kinase; Abs: Antibodies; GAPDH: glyceraldehyde-3-phosphate dehydrogenase; OS: osteogenic differentiation medium; iTRAQ: isotopic multi-tag relative quantitative proteomics; hASCs: human adipose-derived stem cells; SDS-PAGE: sodium dodecyl sulfate polyacrylamide gel electrophoresis; PVDF: polyvinylidene fluoride; ECL: enhanced chemiluminescence; qRT-PCR: quantitative real-time PCR; DMEM: Dulbecco’s modified Eagle medium; DAPI: 4′,6-diamidino-2-phenylindole; BCIP: 3-bromo-4-chloro-3-indolyl phosphate; NBT: nitro blue tetrazolium; ddH_2_O: double distilled H_2_O; FBS: fetal bovine serum; GM: growth medium/undifferentiated; PCA: principal component analysis; Go: gene ontology; CCK-8: Cell Counting Kit-8; ECM: extracellular matrix; TAZ: tafazzin.

**Acknowledgements**

Not applicable

**Availability of data and materials**

All the supporting data can be downloaded. The data presented in this article are available upon request from the corresponding authors.

**Ethics statement**

This animal experiments were approved by the Institutional Animal Care and Use Committee (IACUC) of the Southern Medical University (Resolution NO.: L2018147, Date of Resolution: 09/10/2018).

**Consent for publication**

Not applicable.

**Declaration of competing interest**

The authors declare no conflict of interests.

**Original gel images**

The original gels/blots used in the main figures can be found in Supplemental File2.

**Supplemental Figures**

**Figure S1.** **Identification of PDLIM5 expression related to osteogenic differentiation, cytoskeleton, and cell-matrix condition. (A)** Boxplot shows the difference of PDLIM5 gene expression between osteogenic medium and growth medium in hASCs. **(B)** Principal Component Analysis (PCA) analyzed the dimensional characteristics of PDLIM5 changes in osteogenic differentiation. **(C)** Protein-protein interaction network was constructed for the relationship between PDLIM5 and cytoskeletal actin and cell motility behaviors.

**Figure S2. Surface molecular and related proteins are involved in the osteogenic differentiation of hASCs *in vitro*. (A)** Surface protein markers of CD45, CD24, CD29, CD90, and CD105 were detected by flow cytometry. **(B-C)** Representative immunofluorescence staining and semi-quantitative analysis of PDLIM5 co-localized with α-actinin 1 and F-actin on key stress fibers during osteogenic differentiation in hASCs. Scale bar, 10 μm.

**Figure S3. PDLIM5 and microfilament-related proteins involved in osteogenic differentiation are inhibited by CytoD in hASCs. (A-B)** Representative immunofluorescence staining analysis showed the changes between PDLIM5 and α-actinin 1 and F-actin on key stress fibers during osteogenic differentiation treated with CytoD in hASCs. Scale bar, 10 μm, CytoD: actin polymerization inhibitors.

**Figure S4. PDLIM5 knockdown inhibits the biological behaviors of hASCs. (A)** Gene expression of PDLIM5 by qRT-PCR analysis after lentivirus infection. **(B)** The immunofluorescence staining analysis of PDLIM5 knockdown was detected. Scale bar, 10 μm. **(C)** CCK8 assay results show the proliferation status of the control (shScr) and PDLIM5-silenced (shPDLIM5) hASCs. **(D-E)** PDLIM5 knockdown inhibits hASCs migration and motility. Scale bar, 200 μm. shScr: empty plasmid negative control group, shPDLIM5: an experimental group of PDLIM5 knockdown.

**Figure S5. PDLIM5 knockdown attenuates the osteogenic differentiation of hASCs mediated by microfilaments. (A)** The effect of PDLIM5 knockdown on the morphology of the microfilament skeleton was detected by immunofluorescence at 7 days. **(B)** Immunofluorescence staining showed that PDLIM5 knockdown affected nuclear YAP co-localization of hASCs. Scale bar, 10 μm.

**Figure S6. PDLIM5 knockdown attenuates the transduction of cyclic stretching stimulation during osteogenic differentiation. (A)** Immunofluorescence and semi-quantitative analysis showed that PDLIM5 self-expression decreased after knockdown under cyclic stretching stimulation. **(B)** Under cyclic stretching stimulation, immunofluorescence and semi-quantitative analysis showed that the mechanical sensitive protein YAP was released from the nucleus after PDLIM5 knockdown. Scale bar, 50 μm.

**Figure S7. (A-B)** Immunofluorescence and semi-quantitative analysis showed that the expression of cytoskeletal protein F-actin **(A)** and cytoskeleton-associated protein α-actinin 1 **(B)** decreased after PDLIM5 knockdown under cyclic stretching stimulation. Scale bar, 50 μm.

**Supplemental file1.** The heatmap gene names list.

**Supplemental file2.** The original results of western blotting.
